# Comparison of Antimicrobial Efficacy of Green Tea, Garlic with Lime, and Sodium Fluoride Mouth Rinses against *Streptococcus mutans, Lactobacilli* species, and *Candida albicans* in Children: A Randomized Double-blind Controlled Clinical Trial

**DOI:** 10.5005/jp-journals-10005-1442

**Published:** 2017-02-27

**Authors:** Ann Thomas, Sneha Thakur, Rishika Habib

**Affiliations:** 1 Professor, Department of Pedodontics, A.J. Institute of Dental Sciences Mangaluru, Karnataka, India; 2 Postgraduate Student, Department of Pediatric and Preventive Dentistry, A.J. Institute of Dental Sciences, Mangaluru, Karnataka, India; 3 Postgraduate Student, Department of Pedodontics, A.J. Institute of Dental Sciences Mangaluru, Karnataka, India

**Keywords:** Caries prevention, Herbal mouth wash, Home oral hygiene, Severe early childhood caries.

## Abstract

**Introduction:**

With greater awareness worldwide, the use of herbs and herbal products has increased to a large extent.

**Objective:**

To evaluate and compare the antimicrobial efficacy of green tea, garlic with lime, and 0.05% sodium fluoride (NaF) mouth rinses against *Streptococcus mutans, Lactobacilli* species, and *Candida albicans.*

**Materials and methods:**

A total of 45 children aged 4 to 6 years with severe early childhood caries (S-ECC; based on decayed extracted filled [defs] score) were selected. Children were divided randomly into three equal groups and were asked to rinse with the prescribed mouth rinse once daily for 2 weeks after breakfast under supervision. A base-line and postrinsing nonstimulated whole salivary sample (2 mL) was collected and tested for the number of colony-forming units (CFUs). The data were statistically analyzed using Statistical Package for the Social Sciences (SPSS) version 16.0 software with one-way analysis of variance (ANOVA) and Tukey’s *post hoc* test.

**Results:**

A statistically significant fall in colony count was found with the three mouth rinses in S. *mutans* (p < 0.001, p < 0.001) and *Lactobacilli* spp. (p < 0.001, p < 0.001), but not against *C. albicans* (p = 0.264, p = 0.264). On comparison, no statistically significant difference was found against S. *mutans* (p = 1, p = 0.554, p = 0.572), *lactobacilli* spp. (p = 0.884, p = 0.999, p = 0.819), and *C. albicans* (p = 0.999, p = 0.958, p = 0.983).

**Conclusion:**

The findings of this study indicate that green tea and garlic with lime mouth rinse can be an economical alternative to NaF mouth rinse both for prevention and therapeutics.

**How to cite this article:**

Thomas A, Thakur S, Habib R. Comparison of Antimicrobial Efficacy of Green Tea, Garlic with Lime, and Sodium Fluoride Mouth Rinses against *Streptococcus mutans, Lactobacilli* species, and *Candida albicans* in Children: A Randomized Double-blind Controlled Clinical Trial. Int J Clin Pediatr Dent 2017;10(3):234-239.

## INTRODUCTION

Mouth rinses are extensively promoted in prevention of dental caries. The significance of mouth and teeth cleanliness has been recorded from the ancient days of civilization to the 21st century. The first recognized mention of mouth rinsing is found in Chinese medicine around 2700 **bc**.^1^ Currently, an expansive choice of mouth rinses is available both for preventive and therapeutic purposes against oral diseases.

Dental caries is a preventable, localized transmissible, multifactorial disease resulting from interaction between host, diet, and microflora on the tooth surface over a period of time, resulting in cavitation of inorganic moieties of enamel and dentin.^2,3^ The most commonly related bacteria in its etiology are *S. mutans* for its onset and *Lactobacilli* spp. for its advancement. Off late, a number of reports in the scientific database show an association between *C. albicans,* a fungi, and progression of dental caries.^4^

Of the commercially available mouth rinses, NaF mouth rinse is used for routine home oral hygiene measures. Numerous studies have shown that fluoride not only has an effect on the carbohydrate metabolism by car-iogenic microbes, but also promotes remineralization of a demineralized tooth structure.^5-7^ The NaF has been the compound of choice in various preventive programs.^8-10^ Despite several advantages, there is a fear of ingestion of fluoride in children, as it could lead to fluoride toxicity.^8^

In order to avoid the drawbacks of chemical products, various natural/herbal agents have been launched as mouth rinses. Of the several herbal products being described in the scientific literature, herbs like green tea, garlic, and lime have been used traditionally in effective home remedies.

Tea has been consumed as a beverage for centuries and has demonstrated many health benefits. Green tea is reported to be very rich in fluoride and catechin, a bioac-tive component, which exerts an anticariogenic effect by exhibiting bacteriostatic as well as bactericidal effects on the most commonly implicated bacteria—*S. mutans.^11-13^* The cariostatic activity of catechins present in green tea was found to be related to its role in diminution of thiol group, which, in turn, exercised a bactericidal effect.^13^ It is also known to mediate actions of protective salivary components, such as secretory immunoglobulins, lyso-zymes, lactoferrin, oral peroxidases histatins, mucins, or others, thus exhibiting an indirect anticariogenic effect.^14^

Garlic’s antibacterial activity has been first stated by Louis Pasteur; and there are also reports of its antifungal and antiviral activities.^15,16^ Scientific reports have shown it to have anti-inflammatory and antioxidant property; and also sulfur-containing compounds present in it have known to show an inhibitory effect on *S. mutans.^15-17^* The antimicrobial effect of allicin, the active component of garlic, is due to its reactions with the thiol groups of various cellular enzymes.^17,18^ Due to the presence of allicin and thiosulphonates, it is also suggested that they act in conjugation with antibiotics.^19,20^ It is reported that mouth rinses incorporating garlic prevent the fall in salivary pH, thereby favoring remineralization.^21^ The specific flavor of allicin in garlic induces salivation, and salivary clearance further boosts its anticariogenic effect.^15,21^ Another study that evaluated the antibacterial effect of garlic and lime paste blended together on extracted carious teeth indicated that more clinical studies are required to substantiate its cariostatic effect where lime was incorporated to counteract the pungent flavor of garlic and also for its known antimicrobial activity.^15^

To our knowledge, there is an inadequacy of reports on comparing the antimicrobial efficacy of green tea and garlic with lime mouth rinse to that of NaF, the most widely used mouth rinse; and also, there are no reports regarding its antifungal activity against *C. albicans,* which is recently being linked to the etiology of caries. Thus, the main purpose of this study was to evaluate and compare the antimicrobial efficacies of green tea and garlic with lime mouth rinse with that of NaF (0.05%) against *S. mutans, Lactobacilli* species, *and C. albicans.*

## MATERIALS AND METHODS

A randomized double-blind active controlled clinical trial was conducted at a local preschool from November 2013 to February 2014. The study protocol was reviewed and approved by the Ethical Committee of the institution, and the study was in accordance with The Code of Ethics of the World Medication (Declaration of Helinski) for experiments involving human subjects. A written informed consent was acquired from authorities of the preschool and the parents of the subjects before the onset of the study.

The study was conducted on 45 children aged 4 to 6 years, who were randomly chosen from the preschool. Physically fit children diagnosed with S-ECC according to the definition given by the American Academy of Pediatric Dentistry^22^ were selected for the study. Children who could not expectorate completely or brush their teeth on their own had a definite history of taking antibiotics 3 months before the commencement and during the study period, undergoing orthodontic treatment or with an intraoral prosthesis, had any intraoral pathology, were medically compromised, or for whom parental consent was not given were also not included in the study.

The defs of the children was recorded by means of visible light, mouth mirror, and community periodontal index probe. The sum total of defs was taken into account and based on the caries experience, children were chosen for the study. All the study participants were given a tube of nonfluoridated tooth paste and a tooth brush. The brushing and mouth rinsing technique was shown to all and were advised to brush twice daily. The participants were divided at random into three groups of 15 each by lottery method, i.e., they were asked to pick up chits with the name of the mouth rinse written on them. The children were allotted prenumbered similar mouth rinse bottles and were told to rinse the mouth for 1 minute using 5 mL of the respective mouth rinse daily for 2 weeks in the school. The children rinsed their mouth under the supervision of the principal researcher for 6 working days of the week and on Sundays under parental supervision.

## PREPARATION OF MOUTH RINSES

### Green Tea Mouth Rinse

**Fig. 1:**
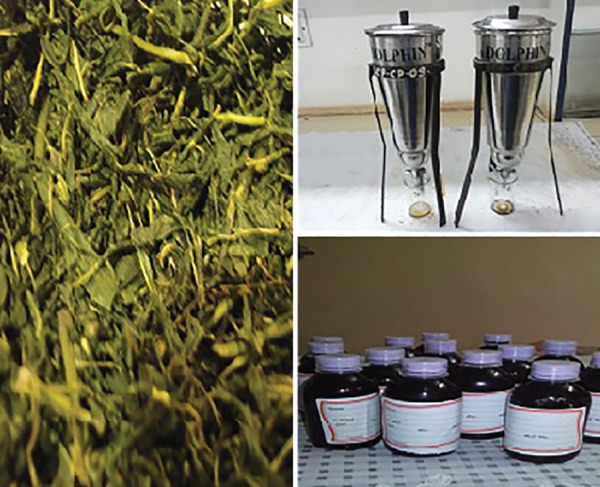
Preparation of green tea mouth rinse

Green tea mouth rinse (Fig. 1) was custom prepared by the pharmacist. To prepare green tea mouth rinse, dried green tea leaves (obtained by open air drying) were grounded to a desirable size using an electrical mill, and then extracted by percolation using distilled water as solvent. Green tea, which is rich in phenolic compounds (6%), was diluted to obtain a concentration of 0.5% phenolic compound using double distilled water. Authorized addi-five, peppermint flavor (1 gm/L), and sodium saccharine (1 gm/L), a sweetening agent, were used to formulate the mouth rinse.^23^

### Garlic with Lime Mouth Rinse

**Fig. 2:**
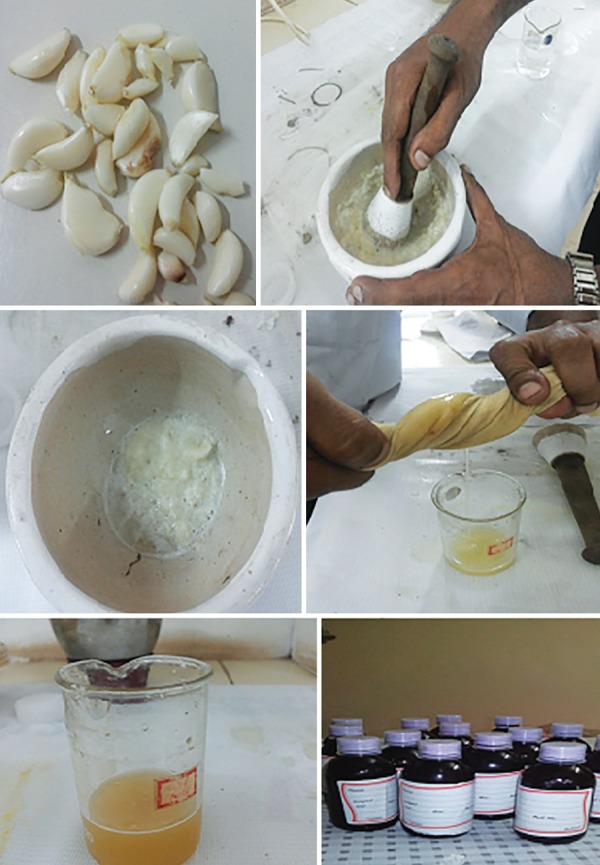
Preparation of garlic with lime mouth rinse

Garlic with lime mouth rinse was custom prepared by the pharmacist (Fig. 2). To prepare garlic with lime mouth rinse, 100 gm of fresh, washed garlic cloves were macerated in a sterile, ceramic mortar and water was added to obtain a homogenate, which was then filtered off with a sterile muslin cloth. The weight of insoluble material was subtracted from the weight of original cloves and the final concentration of the solution was determined to be 1 gm/100 mL. About 100 mL of lime juice was extracted from fresh lemons using a juice extractor and added to the garlic extract. Authorized additive, peppermint flavor (1 gm/L), sodium saccharine (1 gm/L), as sweetening agent, and sodium bicarbonate (0.5 gm) as preservative were added, and the mixture was mixed properly to prepare a mouth rinse.^15^

The NaF was used as a positive control arm (PEPSODENT, Hindustan Unilever).

### Saliva Samples

About 2 mL saliva samples were collected before the commencement of mouth rinsing, i.e., at baseline and after 2 week rinsing, i.e., postrinsing. Unstimulated whole saliva samples were collected by asking the children to drool into a sterile container for 3 to 5 minutes, being seated in an upright position in a bright room with good ventilation. Saliva samples were collected in the morning between 10.00 and 11.00 a.m. in order to avoid any bias in the concentration of saliva due to circadian rhythm.^5^ Children were also told not to eat or drink anything (except water) 1 hour before saliva collection to minimize the possible food debris and stimulation of saliva.

### Microbial Evaluation

**Fig. 3:**
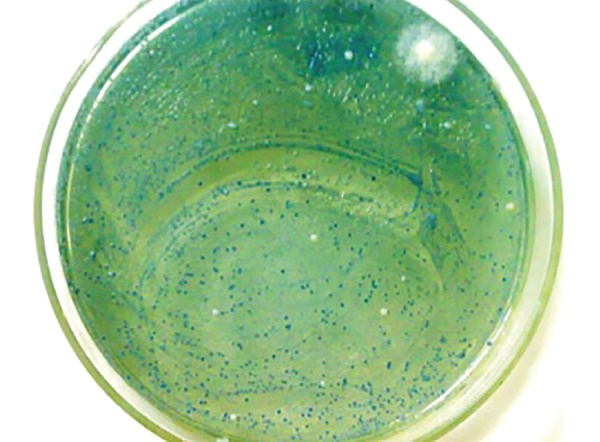
Agar plate with S. *mutans* colonies

**Fig. 4:**
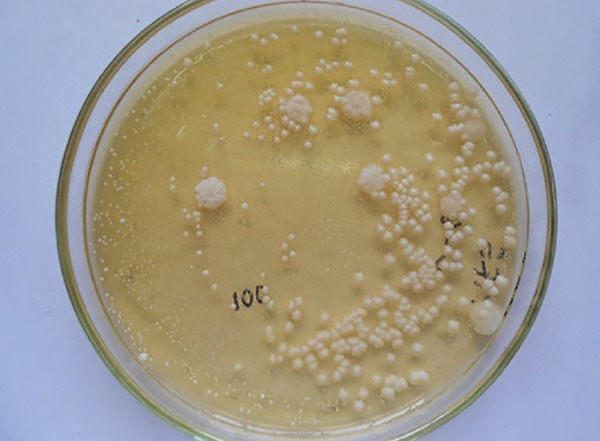
Agar plate with *lactobacilli* colonies

**Fig. 5:**
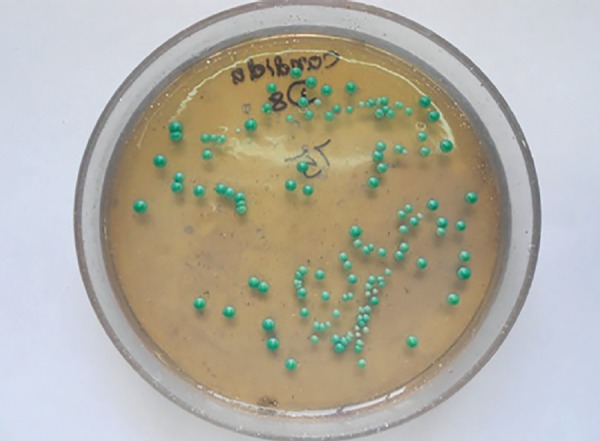
Agar plate with *candida albicans* colonies

The samples were transported to the microbiological laboratory in box with ice packs. They were checked for the CFUs of *S. mutans, Lactobacilli* species, and *C. albicans* using Mitis Salivarius Bacitracin agar, Rogosa agar, and HiChrome agar respectively (HiMedia Laboratories, Mumbai) (Figs 3 to 5). Following serial dilution with physiological saline to obtain minus three [-3] concentrations, 0.1 mL saliva sample was spread on the selective agar plates with a sterile glass spreader. The plates were then incubated for 48 hours at 37°C in the incubation chamber (ROTEK) to get the highest growth of microbial colonies. The CFUs were detected by morphology, size, and color, and counting was done using a hand-held digital colony counter (HiMedia, Mumbai). In order to adjust for the dilution factor, colonies were semiquanti-fied by multiplying the actual colony count with 1 × 10^3^. The colony counting of each plate was carried out thrice by the same observer on different days under constant conditions and in the consistent environmental conditions to avoid the intraobserver variability.

### Evaluation of Acceptability

After the end of the study, the participants were provided with a self-administered, close-ended dichotomous questionnaire to evaluate the acceptability of mouth rinse administered to each of them. The questionnaire included three questions with two mutually exclusive options (YES/ NO) to answer. The questions were based on the acceptability of the mouth rinses in terms of flavor, smell, and willingness to continue using the mouth rinse. A paper and pencil method was used to hand out the questionnaire. With the assistance of the caretaker, the participants’ answer was obtained and the questionnaire was completed.

### Outcome Measures and Statistical Methods

The study had a single endpoint at 2 weeks. The data were statistically analyzed by using one-way ANOVA, Tukey’s *post hoc* honest significant difference (HSD) test respectively, in SPSS software 16.0. The results were considered statistically significant at 0.05 probability level. One-way variance ANOVA test was employed to compare the mean of differential colony counts in the studied mouth rinse groups and assess the antimicrobial efficacy of green tea and garlic with lime mouth rinses, which was the primary outcome of the study. Tukey’s *post hoc* HSD test was used for comparative analysis of the three mouth rinse groups and quantify the secondary outcome, i.e., if the newly formulated green tea and garlic with lime mouth rinses were better than NaF mouth rinse.

**Table 1:** **Table 1:** Mean differential colony counts of S. *mutans, Lactobacilli* species, and *C. albicans* in the three mouth rinse groups

*Mouth rinse group*		*n*		*Microorganism*		*Mean base-line (CFU/mL)*		*Mean postrinse (CFU/mL)*		*Mean difference (CFU/mL)*		*Significance*	
NaF		15		*S. mutans*		4.8 × 10^8^		1.9 × 10^8^		2.9 × 10^8^		p < 0.001	
				*Lactobacilli* spp.		2.5 × 10^5^		0.43 × 10^5^		2.07 × 10^5^		p < 0.001	
				*C. albicans*		7.7 × 10^4^		4.5 × 10^4^		3.2 × 10^4^		p = 0.264	
Garlic with lime		15		*S. mutans*		3.34 × 10^8^		1.6 × 10^8^		1.74 × 10^8^		p < 0.001*	
				*Lactobacilli* spp.		1.83 × 10^5^		0.43 × 10^5^		1.4 × 10^5^		p < 0.001*	
				*C. albicans*		7.1 × 10^4^		4.3 × 10^4^		2.8 × 10^4^		p = 0.264	
Green tea		15		*S. mutans*		2.9 × 108		1.9 × 108		1 × 108		p < 0.001*	
				*Lactobacilli* spp.		4.9 × 104		2.2 × 104		2.7 × 104		p < 0.001*	
				*C. albicans*		6.4 × 104		4.1 × 104		2.3 × 104		p = 0.264	

*Very highly significant (p < 0.001)

## RESULTS

The three studied mouth rinses showed a statistically significant fall in the colony counts of *S. mutans and Lactobacilli* spp. (p < 0.001, p < 0.001), whereas only a numerical fall in *C. albicans* colony count was found, which was not statistically significant (p = 0.264; Table 1).

When the antimicrobial efficacy of the NaF, green tea, and garlic with lime mouth rinses was evaluated, no statistically significant difference was found against *S. mutans* (p = 1, p = 0.554, p = 0.572), *Lactobacilli* spp. (p = 0.884, p = 0.999, p = 0.819), and *C. albicans* (p = 0.999, p = 0.958, p = 0.983) (Table 2).

Majority of the study participants in the NaF group had a positive response to flavor (n = 10; 66.7%), smell (n = 10; 66.7%), and willingness to rinse (n = 11; 73.3%). The response of the study participants in the green tea group indicated that majority of the study participants in the green tea group had a positive response to flavor (n = 10; 66.7%), smell (n = 8; 55.3%), and willingness to rinse (n = 10; 66.7%), whereas the results for the garlic with lime mouth rinse group gave a negative response for flavor and smell (n = 10; 66.7%; n = 10; 66.7%). The response for willingness to continue rinsing was found to be mediocre (n = 8; 53.3%).

**Table 2:** **Table 2:** Intercomparison of the mean difference of colony counts of the three mouth rinse groups for S. *mutans,* *Lactobacilli* species, and *C. albicans*

*Dependent variable*		*(I) group*		*(J) group*		*Mean difference (I-J) 10^3^ (CFU/mL)*		*Std. error*		*p-value*	
Diff. *S. mutans*		NaF		Green tea		1733.333		59868.01		1	
				Garlic with lime		79066.67		59868.01		0.554	
		Green tea		Garlic with lime		77333.33		59868.01		0.572	
Diff. *Lactobacilli* spp.		NaF		Green tea		36.867		50.431		0.884	
				Garlic with lime		–7.133		50.431		0.999	
		Green tea		Garlic with lime		–44		50.431		0.819	
Diff. *C. albicans*		NaF		Green tea		–10.267		72.176		0.999	
				Garlic with lime		–36.467		72.176		0.958	
		Green tea		Garlic with lime		–26.2		72.176		0.983	

## DISCUSSION

The purpose of this study was to evaluate and compare the antimicrobial efficacy of green tea and garlic with lime mouth rinses with that of NaF mouth rinse on the level of salivary *S. mutans, Lactobacilli* species, *and C. albicans* in children. The present study was carried out under real-life conditions without altering the subjects’ routine oral hygiene practices except that they were advised to use a nonfluoridated dentifrice for cleaning their teeth. This was done to eliminate the bias resulting due to additional antimicrobial effect of fluoride from the dentifrice. To standardize rinsing, all the study participants were asked to rinse under monitoring at school. Thus, rinsing was carried out after breakfast and this could have also contributed toward reducing the microbial challenge.

According to the results of this study, green tea was found to be a very effective antibacterial mouth rinse against *S. mutans and Lactobacilli* spp. with some antifungal activity against *C. albicans.* The antibacterial effect of green tea mouth rinse is in accordance to the previous reports where the authors stated that rinsing with green tea extract had valuable anticariogenic activities including inhibitory effect on cariogenic bacteria by inhibiting the adherence of bacterial cells to the tooth surfaces.^13,23-25^ And also, green tea catechins maintain the salivary pH at a normal range, which is not a favorable condition for cariogenic bacteria to flourish.^26,27^ The antifungal activity is in agreement to the earlier *in vitro* studies that have reported that green tea polyphenols and catechins inhibit the growth of *C. albicans* by 40 and 75% respectively.^26-28^

*In vitro* and *in vivo* data have revealed that garlic extract could significantly inhibit the growth of many bacteria, fungi, and viruses.^15-17,21,29^ The antibacterial activity of garlic with lime mouth rinse is in accordance to the earlier *in vivo* studies^7,8,30^ and *in vitro* studies.^15,17,21,29^ The antifungal activity is in agreement with the earlier *in vitro* studies, which reported that pure allicin, which is also an active component of garlic, to be effective against many fungi due to its inhibitory function on thiol enzymes.

In this study, no significant difference in the antibacterial efficacies of green tea and NaF mouth rinses against *S. mutans and Lactobacilli* spp. was found; and this finding is in agreement to a previous report.^23^

When the antimicrobial efficacy of garlic with lime mouth rinse was compared with that of NaF mouth rinse, no significant difference was found against the three studied microbes. To compare our findings with the previous reports, our literature search revealed absence of reports in this area. Our study also revealed that green tea and garlic with lime mouth rinses were comparable to each other in their antimicrobial activity against the three studied microbes.

The study participants’ acceptance of the prescribed mouth rinse was evaluated using a questionnaire. The reduced tolerance for flavor and smell of garlic with lime mouth rinse has been documented in a previous study where the authors specified that the possible reason for this could be due to the burning sensation caused by allicin.^30^ The acceptance for green tea and NaF mouth rinses in terms of flavor, smell, and willingness to continue rinsing was good.

## CONCLUSION

From the results of our study, it can be concluded that green tea and garlic with lime mouth rinses could be very good cost-effective alternatives to NaF mouth rinse. However, further studies would be beneficial to evaluate any potential adverse effects with long-term use of these mouth rinses.
